# COVID-19 vaccine hesitancy in the UK: a longitudinal household cross-sectional study

**DOI:** 10.1186/s12889-021-12472-3

**Published:** 2022-01-15

**Authors:** Kausik Chaudhuri, Anindita Chakrabarti, Joht Singh Chandan, Siddhartha Bandyopadhyay

**Affiliations:** 1 Economics Division, Leeds University Business School, Leeds, LS2 9JT England; 2 Institute of Applied Health Research, University of Birmingham, Birmingham, B152TT England; 3 Department of Economics, Birmingham Business School and Centre for Crime Justice and Policing, University of Birmingham, Birmingham, B15 2TT England

**Keywords:** COVID-19, SARS-CoV-2, Vaccine hesitancy, Ethnicity

## Abstract

**Background:**

The approved COVID-19 vaccines have shown great promise in reducing disease transmission and severity of outcomes. However, the success of the COVID-19 vaccine rollout is dependent on public acceptance and willingness to be vaccinated. In this study, we aim to examine how the attitude towards public sector officials and the government impact vaccine willingness. The secondary aim is to understand the impact of ethnicity on vaccine-willingness after we explicitly account for trust in public institutions.

**Methods:**

This cross-sectional study used data from a UK population based longitudinal household survey (Understanding Society COVID-19 study, Understanding Society: the UK Household Longitudinal Study) between April 2020-January 2021. Data from 22,421 participants in Waves 6 and 7 of the study were included after excluding missing data. Demographic details in addition to previous survey responses relating to public sector/governmental trust were included as covariates in the main analysis. A logit model was produced to describe the association between public sector/governmental mistrust and the willingness for vaccination with interaction terms included to account for ethnicity/socio-economic status.

**Results:**

In support of existing literature, we identified those from BAME groups were more likely to be unwilling to take the COVID-19 vaccine. We found that positive opinions towards public sector officials (OR 2.680: 95% CI 1.888 – 3.805) and the UK government (OR 3.400; 95% CI 2.454—4.712) led to substantive increase in vaccine willingness. Most notably we identified this effect to vary across ethnicity and socio-economic status with those from South Asian background (OR 4.513; 95% CI 1.012—20.123) and possessing a negative attitude towards public officials and the government being the most unwilling to be vaccinated.

**Conclusions:**

These findings suggests that trust in public sector officials play a key factor in the low vaccination rates particularly seen in at-risk groups. Given the additional morbidity/mortality risk posed by COVID-19 to those from lower socio-economic or ethnic minority backgrounds, there needs to be urgent public health action to review how to tailor health promotion advice given to these groups and examine methods to improve trust in public sector officials and the government.

## Background

The COVID-19 pandemic has posed major challenges to public health systems across the world including the four major nations in Europe: Italy, Spain, France, and the United Kingdom (UK) which struggled to cope with the increasing deaths and hospitalisation due to the disease, partly because of its already over-burdened health system [1]. At the same time, there was a concerted global effort to develop and deliver safe and efficacious vaccinations at record speed. The UK has been a leading country during the vaccine rollout as more than three quarters of the population received at least one dose by early June 2021 [2]. By early April, Public Health England estimated that the vaccines had prevented over 10,000 deaths in people over the age of sixty and in Israel, initial evidence demonstrated a marked reduction in the Sars-CoV-2 infection rate and related morbidity and mortality due to vaccination. [3]

Despite the evidenced efficacy, concerns have been raised around existing hesitancy to accept the vaccine which may put the success of the public health initiative at risk [4]. The World Health Organization (WHO) strategic advisory group of experts (SAGE) defines vaccine hesitancy as “a delay in acceptance or refusal of vaccination despite availability of vaccination services” and hence caters to a wider audience – not just people who refuse to be vaccinated [5]. Within the UK by the 18 February 2021, 34% of 18,855 participants of OCEANS II/III study confirmed that they were either doubtful or strongly unwilling to opt for the vaccine [6]. Similar mistrust was also noted amongst a higher risk category namely the ‘keyworkers’ with 23.9% of the 579 keyworkers surveyed responding that they are uncertain or will refuse to take the vaccine [7]. These rates of COVID-19 vaccine hesitancy have remained relatively stable since earlier studies conducted by January 2021 suggesting around 50–60% of individuals would be willing to receive a vaccine [8, 9]. These findings are in line with a recent systematic review collating information on all validated cross-sectional surveys examining vaccine hesitancy [10]. This review identified 53 studies from Europe alone, identifying similar rates of vaccine hesitancy to the UK in countries such as Italy, Poland, France, Germany and Greece [10–14]. When examining the UK data, 1) those of Black and ethnic minority background and 2) lower socioeconomic households or those currently unemployed, have indicated even greater rates of hesitancy [4]. Such findings corroborates results from the US and France [15–17]. Concerningly, these groups are at greater risk of transmission of COVID-19 as well as subsequent morbidity and mortality. [18].

Where authors have hypothesised the rationale for the hesitancy in these at-risk groups, uniform messages emanate: mistrust in the vaccine may correlate with mistrust in public sector governing/health bodies [4, 6, 19]. However, this feature has not been explicitly examined within the existing literature, particularly in terms of formal statistical analysis.

Therefore, in this study we aim to examine how one’s attitude towards public sector officials and the government impacts vaccine willingness. The secondary aim is to understand the impact of ethnicity on vaccine-willingness after we explicitly account for trust in public institutions.

## Methods

### Study design and data

This study used data from a UK population based longitudinal household survey (Understanding Society COVID-19 study, Understanding Society: the UK Household Longitudinal Study) between April 2020-January 2021. [20].

The samples are probability samples of postal addresses with slight variations in how the sampling was done across England, Wales and Scotland vs Northern Ireland. In England, Wales and Scotland they are clustered and stratified whereas in Northern Ireland they are unclustered, systematic random samples. Northern Ireland and areas in Great Britain with high immigrant and ethnic minority populations were oversampled. The survey consists of all eligible consenting individuals aged 16 years and over in eligible households. The survey was undertaken monthly between April-July 2020 and then two monthlies thereafter, with only those who had completed prior surveys eligible for continued entry in the latter months [21]. The full details for this survey have been described in depth previously. [21].

### Inclusion criteria for Outcome data

Although the survey collected data on numerous indicators of clinical and social status, in this study we only included those who had been administered/completed the flu and coronavirus vaccine questions in both wave 6 (November 2020) and wave 7 (in January 2021) of the Covid-19 survey. The criteria for being included in both waves was that in wave 7 only those respondents were eligible who were yet to receive the actual vaccination or an invitation for it. Note, the wave 6 survey asked respondents “Imagine that a vaccine against COVID-19 was available for anyone who wanted it. How likely or unlikely would you be to take the vaccine?” The respondent answers options are “very likely, likely, unlikely and very unlikely”. Wave 7 asks the respondents the same question however only to those who had not yet received a COVID vaccine or an appointment for one. In wave 6, a total of 12,035 respondents were eligible but 80 were excluded from our analysis as they selected one of the missing/inapplicable/refusal/don’t know responses. For wave 7, out of 11,968 respondents, 23 respondents were excluded due to missing/inapplicable/refusal and 2861 respondents were excluded because they had received at least one dose of vaccination and / or invitation for it (2,109 respondents had received the 1^st^ dose, 148 both and 604 respondents received an invitation for vaccination). Combining the two waves (wave 6 and wave 7), we were left with 23,572 respondents for our outcome variable: vaccine willingness.

### Predictor Variables

Although, we only included respondents active and eligible during wave 6 (November 2020) and wave 7 (in January 2021) of the Covid-19 survey, we utilised covariate data also from the previous waves (for example wave 9 main survey conducted in 2018/19) to track individual respondents over time. Data were taken from previous waves of the COVID-19 survey as well as iterations of the UK Household survey. Doing so provided the following demographic data for use in this study respondents as follows: 1) age, 2) gender and marital status, 3) ethnicity as per UK census definitions [22], 4) attained educational qualifications, 5) employment status, 6) household living arrangements, 7) clinical vulnerability, 8) subjective financial condition, 9) household monthly current income for sensitivity analysis and 10) geographical region. In addition we controlled for the time when the survey was conducted. Additionally, in the wave 9 main survey conducted in 2018/19,[21] respondents were asked the following questions: “Public officials don't care” and “Don't have a say in what government does”. For both, the respondents’ answers could be **“**strongly agree/agree, neither agree/disagree, strongly disagree/disagree.” These questions were included in our final dataset as indicators of public sector official and government trust.

We use disaggregated ethnicity: South Asian (Indian, Bangladeshi, Pakistani), Black (Black African, Black Caribbean, Other Black), any other Asian, Mixed (White and Black Caribbean, White and Black African, white and Asian), and other (Arab/any other) with White as the reference category. We include gender (male taking the value of 1), age, educational qualification (degree, A- level/post-secondary, GCSE (General Certificate of Secondary Education), basic (lower than GCSE) with no formal qualification as the base category), employment status (employed, self-employed, both employed & self-employed and none/retired), living arrangement (living with household members aged 70 or older, excluding respondent), clinically vulnerable as identified by NHS (no risk, moderate/high risk), and current subjective financial condition (comfortable/alright, same, and bad). For variables representing markers of public sector official and government trust, the respondents’ answers are “strongly agree/agree, neither agree/disagree, strongly disagree/disagree” (excluding missing/refusal). We use these two variables to create three binary indicators: positive (strongly disagree/disagree), neutral (neither agree/disagree) and negative (strongly agree/agree) and use two of them (positive and neutral) with negative as the base category.

### Missing data

Recorded ethnicity was missing for 540 respondents (255 for wave 6 and 285 for wave 7) and these individuals were excluded from our analysis. Ten respondents from each wave were also excluded due to no recorded sex. Educational qualifications were missing for 86 respondents in wave 6 and 82 for wave 7 of COVID -19 survey, who were also excluded. as were 14 respondents, geographical location of their residence was unavailable. Further, respondents with missing/inapplicable observations for variables representing as markers of public sector official and government trust were also excluded leaving us with 22,421 observations.

### Statistical analysis

The primary outcome data taken from responses in wave 6 (November 2020) and wave 7 (in January 2021) of the COVID-19 study were re-classified to a vaccine willingness category taking the value of one if the respondents gave a positive response (very likely, likely) and 0 if unlikely and very unlikely. For our combined model, we pooled the data on vaccine willingness category from both rounds. Covariate data were also transformed into binary or categorical groups depending on the nature of the data.

We analysed the data using a logit model where the dependent variable (vaccine willingness:) taking the value of 1 and 0 otherwise as follows:

$$\mathrm{log}\left(\frac{{Prob(vaccine willingness}_{it}=1)}{Prob\left({vaccine wiilingness}_{it}=0\right)}\right)={\upbeta }_{0}+{\sum }_{i}^{k}{\upbeta }_{i}{x}_{i,k,t}+\sum_{r=1}^{11}{\alpha }_{r}{+ e}_{it}$$

where ${x}_{i,k,t}$ represents covariates/predictor variables for individual *i* in wave *t* and ${\alpha }_{r}$ is the binary indicator for respondents’ region of residence (there are 12 regions and we use North-East as the reference category). Regression coefficients (${\beta }_{i}$) were exponentiated and are presented as odds ratios (OR) with corresponding exponentiated 95% confidence intervals (CI) and the *p*-values (*p*) for significance with *p* < 0·10 considered to indicate statistical significance. We use robust standard errors to account for some types of misspecification and allowing for intra-class correlation at the individual level. For the main analyses we analysed two models: Model 1 includes the attitude of respondents towards public officials and Model 2 includes whether respondents felt they had a say in government. We also ran logit models with interaction terms where we interact the attitude of respondents towards public officials/ say in government with ethnicity variable (Model 3/Model 4). We use likelihood ratio tests for the interaction model to examine whether their opinion about public officials/government significantly affects the decision of minority communities to get vaccinated. Models were estimated using Stata 15.

## Results

### Study characteristics

During the study period, 22,421 respondents were eligible to be included in the final study. Of these, the average age was 55 (SD 15.5) with the majority being female (13,112/22421 (59.5%). Most respondents were White (20,439/22421 (91%)) and in terms of educational status 15,878/22421 (71%) were educated to an A-level standard or higher. Although, 10,500/22421 (47%) of respondents were currently employed, 16,792/22421 (75%) felt financially comfortable/alright. Variables regarding opinion about public officials and caring by government reveal that 4423/22421 (19.7%) and 6012/22424 (26.8%) show positive attitude whereas 8851/22421 (39.5%) and 8084/22424 (36%) display negative attitude. The study characteristics of the sample are presented in Table 1.

**Table 1:** Summary Statistics

Variable	Proportion/Mean	SD
Age	55.465	15.448
Gender		
Male	9309 (0.415)	0.493
Female	13,112 (0.585)	0.493
Marital Status		
Couple	15,966 (0.712)	0.453
Ethnicity		
South Asian	931 (0.042)	0.200
Any other Asian	226 (0.010)	0.100
Black	398 (0.018)	0.132
Mixed	320 (0.014)	0.119
White & Other (ref.)	20,546 (0.916)	0.277
Educational Qualifications		
No qualification dummy (ref.)	963 (0.043)	0.203
Basic qualification dummy	1585 (0.071)	0.256
GCSE qualification dummy	3995 (0.178)	0.383
A-level qualification dummy	7762 (0.346)	0.476
Degree dummy	8116 (0.362)	0.481
Employment Status		
Employed dummy (ref.)	10,500 (0.468)	0.499
Self-employment dummy	1685 (0.075)	0.264
Both Employed/self-employed dummy	447 (0.020)	0.140
Retired/others dummy	9789 (0.437)	0.496
Household Living Arrangement		
No member aged equal/above 70 (ref)	18,692 (0.834)	0.372
At least 1 member aged equal/above 70	3729 (0.166)	0.372
Clinically Vulnerable Dummy	9914 (0.442)	0.497
Subjective Financial Condition		
Finance current same as before (ref)	4665 (0.150)	0.357
Finance current alright/comfortable	16,792 (0.749)	0.434
Finance current bad/very bad	964 (0.043)	0.203
Opinion about Public Official		
Negative opinion (ref)	8851 (0.395)	0.489
Neutral opinion	9147 (0.408)	0.491
Positive opinion	4423 (0.197)	0.398
Opinion about Public Official		
Negative opinion (ref)	8082 (0.360)	0.480
Neutral opinion	8328 (0.371)	0.483
Positive opinion	6014 (0.268)	0.443

### Main findings

**Fig. 1:**
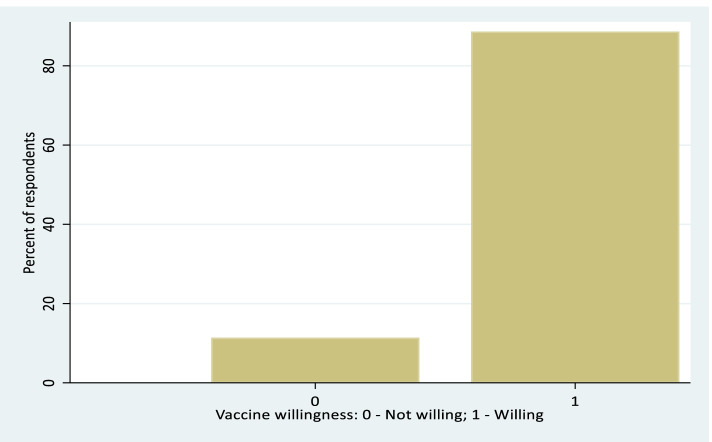
Vaccine Willingness (in percent)

During the study period we found that 19,915/22241 (88.8%) of the respondents were willing to take the vaccine (Fig. 1).

Starting with our focused variables, we find that respondents from ethnic minority groups shows a varied pattern: Black [OR: 0.004, CI: 0.002—0.010, *p*-value < 0.00 for Model 1 & 2] are least willing, followed by South Asian [OR: 0.106, CI: 0.064—0.176, *p*-value < 0.00 for Model 1 & OR: 0.110, CI: 0.066—0.183, *p*-value < 0.00 for Model 2 respectively]. Note, Black, Asian and Minority Ethnic (BAME) populations are at an increased risk of developing COVID-19 and consequentially more severe outcomes compared to White populations.

**Fig. 2:**
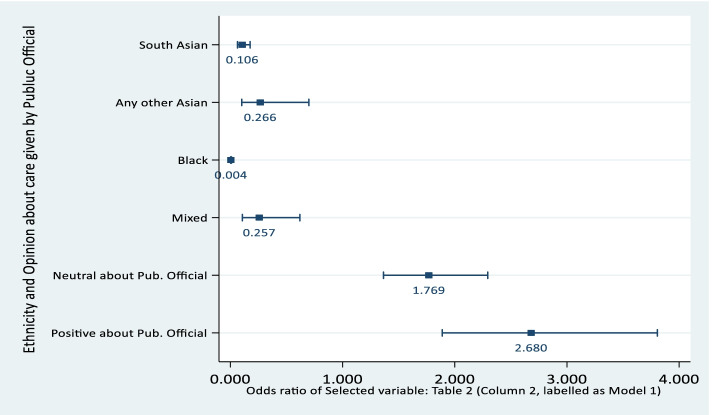
Ethnicity and Opinion about Care given by Public Official

**Fig. 3:**
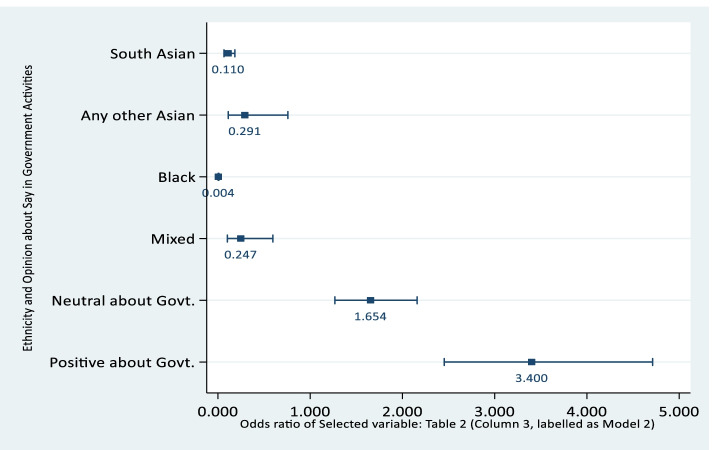
Ethnicity and Opinion about Say in Government Activities

Positive/Neutral opinion whether public officials care increase the odds of vaccine willingness [OR: 1.769, *p*-value < 0.00 (for neutral), and OR: 2.680, *p*-value < 0.00 (for positive), in case of Model 1]. The same correlation with vaccine willingness is corroborated when examining whether respondents felt they had a say in government instead of looking at attitude towards public officials (Model 2) - see Figs. 2 and 3.

Regarding other predictor variables, we found that vaccine willingness is positively and significantly associated with age (OR = 1.079 (CI = 1.069—1.089) in model 1 and 1.077 (1.067—1.087) in model 2). People with lower levels of education are more likely to be unwilling to take the vaccine whereas the clinically vulnerable respondents are more willing [OR: 1.359, CI: 1.046—1.765, *p*-value = 0.02 for Model 1; OR: 1.361, CI: 1.047—1.769, *p*-value = 0.02 for Model 2]. Males and couples are more inclined to get vaccinated compared to females and single people. Self-employed are less willing to get vaccinated [OR: 0.471, CI: 0.310—0.716, *p*-value < 0.00 for Model 1; OR: 0.472, CI: 0.310—0.718, *p*-value < 0.00 for Model 2] compared to employed people (base category). Respondents living with a 70-plus aged member are more willing to get vaccinated. The odds of vaccine willingness for respondents with better subjective financial well-being is 2.758 (Model 1) and 2.692 (Model 2) times that of respondents with the same objective financial condition; whereas the willingness is significantly lower for financially worse off respondents (OR = 0.493 (CI = 0.289—0.840) for Model 1 and OR = 0.504 (CI = 0.296—0.861) for Model 2).

Next in Table 2, we examine whether vaccine willingness by ethnic minority differs for those respondents who do not have a negative opinion about whether public officials/government care. We observe that all the variables retain their sign and significance as in our original model (Table 3) and respondents belonging to ethnic minority without negative opinion about whether public officials care are more willing to take vaccines. The impact is significant for two groups, for example, South Asian showing positive attitude [OR: 4.513, CI: 1.012—20.123, *p*-value = 0.05] are 4.513 times and respondents belonging to Mixed ethnicity with neutral opinion [OR: 11. 11.958, CI: 1.696—84.337, *p*-value = 0.01] are 11.958 times more willing to get vaccinated compared to the White/Other ethnicity group demonstrating neutral/negative attitude. However, the interaction term between trust and ethnicity is positive for each of the four minority groups.

**Table 2:** Logit regression predictors of Vaccine Willingness, Interaction Model

	Model 3 OR (*p*-val) [CI]	Model 4 OR (*p*-val) [CI]
Age	1.078^c^	1.078^c^
	(0.00)	(0.00)
	[1.068—1.089]	[1.067—1.088]
Financial Condition		
Finance same (ref. category)	-	-
Finance current alright/comfortable	2.761^c^	2.705^c^
	(0.00)	(0.00)
	[2.096—3.637]	[2.052—3.566]
Finance current bad/very bad	0.496^b^	0.516^b^
	(0.01)	(0.02)
	[0.291—0.846]	[0.301—0.883]
Resp Couple or not	1.633^c^	1.643^c^
	(0.00)	(0.00)
	[1.267—2.105]	[1.274—2.119]
Male Respondent	2.403^c^	2.435^c^
	(0.00)	(0.00)
	[1.870—3.090]	[1.892—3.133]
Ethnicity		
White & others (ref. category)	-	-
South-Asian	0.060^c^	0.113^c^
	(0.00)	(0.00)
	[0.026—0.140]	[0.048—0.267]
Any other Asian	0.096^c^	0.097^c^
	(0.01)	(0.01)
	[0.017—0.544]	[0.018—0.524]
Black	0.003^c^	0.003^c^
	(0.00)	(0.00)
	[0.001—0.013]	[0.001—0.010]
Mixed	0.071^c^	0.090^c^
	(0.00)	(0.00)
	[0.017—0.295]	[0.018—0.438]
Clinically Vulnerable	1.354^b^	1.368^b^
	(0.02)	(0.02)
	[1.042—1.759]	[1.051—1.780]
Education		
No qualification (ref. category)	-	-
Basic qualification	1.361	1.376
	(0.40)	(0.38)
	[0.668—2.776]	[0.673—2.812]
GCSE qualification	2.376^c^	2.372^c^
	(0.01)	(0.01)
	[1.262—4.472]	[1.258—4.473]
Alevel/post-secondary	3.190^c^	3.083^c^
	(0.00)	(0.00)
	[1.725—5.897]	[1.664—5.712]
Degree	10.995^c^	10.346^c^
	(0.00)	(0.00)
	[5.828—20.744]	[5.472—19.564]
Employment Status		
Employed (ref. category)	-	-
Self-employment	0.464^c^	0.462^c^
	(0.00)	(0.00)
	[0.305—0.704]	[0.303—0.704]
Both Employed/self-employed	0.661	0.652
	(0.28)	(0.27)
	[0.311—1.405]	[0.306—1.388]
Retired/others	1.086	1.095
	(0.58)	(0.54)
	[0.812—1.453]	[0.819—1.466]
HH with atleast 1 member equal/above 70	3.592^c^	3.693^c^
	(0.00)	(0.00)
	[2.172—5.939]	[2.232—6.111]
Opinion about Public Official		
Negative opinion abt. public official (ref. category)	-	-
Neutral opinion abt. Public official	1.545^c^	
	(0.00)	
	[1.171—2.038]	
Positive opinion abt. Public official	2.210^c^	
	(0.00)	
	[1.533—3.185]	
Neutral opinion abt. Public official^a^South-Asian	2.165	
	(0.15)	
	[0.757—6.189]	
Neutral opinion abt. Public official^a^Any Other Asian	3.863	
	(0.23)	
	[0.432—34.565]	
Neutral opinion abt. Public official^a^Black	1.462	
	(0.66)	
	[0.268—7.988]	
Neutral opinion abt. Public official^a^Mixed	11.958^b^	
	(0.01)	
	[1.696—84.337]	
Positive opinion abt. Public official^a^South-Asian	4.513^b^	
	(0.05)	
	[1.012—20.123]	
Positive opinion abt. Public official^a^Any Other Asian	10.958	
	(0.15)	
	[0.407—295.033]	
Positive opinion abt. Public official^a^Black	2.992	
	(0.36)	
	[0.289—30.953]	
Positive opinion abt. Public official^a^Mixed	6.775	
	(0.15)	
	[0.514—89.350]	
Opinion about Govt		
Negative opinion abt. say in Govt. (ref. category)	-	-
Neutral opinion abt. Govt		1.543^c^
		(0.00)
		[1.161—2.050]
Positive opinion abt. Govt		3.201^c^
		(0.00)
		[2.261—4.531]
Neutral opinion abt. Govt.^a^South-Asian		1.041
		(0.94)
		[0.352—3.082]
Neutral opinion abt. Govt^a^Any other Asian		4.247
		(0.19)
		[0.482—37.389]
Neutral opinion abt. Govt^a^Black		2.198
		(0.38)
		[0.373—12.970]
Neutral opinion abt. Govt^a^Mixed		4.121
		(0.18)
		[0.511—33.208]
Positive opinion abt. Govt.^a^South-Asian		0.687
		(0.59)
		[0.176—2.679]
Positive opinion abt. Govt^a^Any other Asian		15.178^a^
		(0.09)
		[0.644—357.730]
Positive opinion abt. Govt.^a^Black		2.191
		(0.43)
		[0.308—15.589]
Positive opinion abt. Govt.^a^Mixed		5.910
		(0.15)
		[0.535—65.282]
*N*	22,421	22,424

Based on robust standard error adjusting for clustering at the individual level. ^a^*p* < 0.1; ^b^*p* < 0.05; ^c^*p* < 0.01

**Table 3:** Logit regression predictors of Vaccine Willingness

	Model 1 OR (*p*-val) [CI]	Model 2 OR (*p*-val) [CI]
Age	1.079^c^	1.077^c^
	(0.00)	(0.00)
	[1.069—1.089]	[1.067—1.087]
Financial Condition		
Finance same (ref. category)	-	-
Finance current alright/comfortable	2.758^c^	2.692^c^
	(0.00)	(0.00)
	[2.094—3.634]	[2.043—3.546]
Finance current bad/very bad	0.493^c^	0.504^b^
	(0.01)	(0.01)
	[0.289—0.840]	[0.296—0.861]
Resp Couple or not	1.648^c^	1.635^c^
	(0.00)	(0.00)
	[1.278—2.124]	[1.269—2.106]
Male Respondent	2.411^c^	2.401^c^
	(0.00)	(0.00)
	[1.876—3.099]	[1.868—3.086]
Ethnicity		
White & others (ref. category)	-	-
South-Asian	0.106^c^	0.110^c^
	(0.00)	(0.00)
	[0.064—0.176]	[0.066—0.183]
Any other Asian	0.266^c^	0.291^b^
	(0.01)	(0.01)
	[0.101—0.700]	[0.112—0.757]
Black	0.004^c^	0.004^c^
	(0.00)	(0.00)
	[0.002—0.010]	[0.002—0.010]
Mixed	0.257^c^	0.247^c^
	(0.00)	(0.00)
	[0.107—0.620]	[0.102—0.596]
Clinically Vulnerable	1.359^b^	1.361^b^
	(0.02)	(0.02)
	[1.046—1.765]	[1.047—1.769]
Education		
No qualification (ref. category)	-	-
Basic qualification	1.372	1.376
	(0.38)	(0.38)
	[0.673—2.797]	[0.675—2.803]
GCSE qualification	2.403^c^	2.352^c^
	(0.01)	(0.01)
	[1.276—4.526]	[1.251—4.423]
Alevel/post-secondary	3.235^c^	3.056^c^
	(0.00)	(0.00)
	[1.749—5.984]	[1.654—5.646]
Degree	11.154^c^	10.180^c^
	(0.00)	(0.00)
	[5.908—21.056]	[5.400—19.190]
Employment Status		
Employed (ref. category)	-	-
Self-employment	0.471^c^	0.472^c^
	(0.00)	(0.00)
	[0.310—0.716]	[0.310—0.718]
Both Employed/self-employed	0.655	0.671
	(0.27)	(0.30)
	[0.309—1.390]	[0.315—1.430]
Retired/others	1.093	1.091
	(0.55)	(0.56)
	[0.818—1.462]	[0.816—1.458]
HH with atleast 1 member equal/above 70	3.680^c^	3.616^c^
	(0.00)	(0.00)
	[2.220—6.098]	[2.198—5.949]
Opinion about Public Official		
Negative opinion abt. public official (ref. category)	-	-
Neutral opinion abt. Public official	1.769^c^	
	(0.00)	
	[1.364—2.294]	
Positive opinion abt. Public official	2.680^c^	
	(0.00)	
	[1.888—3.805]	
Opinion about Govt		
Negative opinion abt. say in Govt. (ref. category)	-	-
Neutral opinion abt. say in Govt		1.654^c^
		(0.00)
		[1.267—2.160]
Positive opinion abt. say in Govt		3.400^c^
		(0.00)
		[2.454—4.712]
*N*	22,421	22,424

Based on robust standard error adjusting for clustering at the individual level. ^a^*p* < 0.1; ^b^*p* < 0.05; ^c^*p* < 0.01

### Sensitivity Analysis

Results reported in Appendix 1 augments the model presented in Table 2 by including the monthly income variable. Instead of using income as a continuous variable, we use quartile dummies for income variable (quartile 1 is the lowest and quartile 4 the highest) after adjusting for household size. The odds ratio of all variables along with their significance remains unaltered as reported in Table 2. Higher levels of household monthly income were associated with more willingness to take vaccines.

Appendix 2 presents the results disaggregated by age categories (16–34, 34 -49, 50 -64 and 65 plus). The findings show that the odds against vaccine willingness decreases with age. Respondents belonging to the younger age group without negative opinion about whether public officials care, do not exhibit greater willingness to take vaccines. However, people belonging to ethnic minority (combined category of South-Asian/Any Other Asian, Black and Mixed) in the younger age groups not having negative attitude about whether public officials care are more willing to take the vaccine with the impact becoming significant for age-group 34–49. For example, ethnic minority in this age-group showing positive attitude [OR: 10.895, CI: 2.239—53.009, *p*-value < 0.00] are almost 11 times more willing to get vaccinated. The same qualitative finding is obtained with the variable representing respondents’ opinion about government.

## Discussion

In summary we found that COVID-19 vaccine willingness varied substantially based on individual demographics and personal opinions about public sector officials/government. We found that vaccine hesitancy is associated with younger age, being female, not living as couple, lower educational level and income, bad financial subjective wellbeing, belonging to BAME community, and in those who are self-employed. In contrast, clinically vulnerable individuals and households with adults aged 70 or more, portray higher vaccine willingness. It was clear that apart from these demographic factors, either positive or negative opinions about public officials/government were also associated with vaccine willingness.

Given the fact that those ethnic minority populations face a higher risk of mortality from COVID, as do those who are from areas with higher levels of deprivation, we might have expected this increased risk to correlate with a higher demand for vaccination in these groups which was not seen in our findings [18, 23]. While differences in individual risk perceptions has been identified as a significant determinant of vaccine decisions worldwide, individual’s perceptions of risk depends crucially on the ability to process information and is not always related to objective risk. Given the uncertainty surrounding the pandemic and human limitations of processing information—people use mental shortcut / heuristics to form their opinion [24, 25]. Thus, apart from knowledge / information on the available vaccines, such heuristics / biases play a key role and social norms i.e. behavioural rules within an ethnic group may be a deciding factor in shaping these biases/ heuristics. Vaccine- uptake judgments thus will depend on other factors apart from public information. Further, how much of public information released by the state is believed depends on citizen’s trust levels and there is no reason to take trust in public authorities to be the same across different sections of the population groups. We identify a crucial factor, namely trust in government which might explain why the BAME population have lower vaccination takeup, something that has been noted as a factor in earlier studies based on a qualitative analysis [26, 27]. Many in these ethnic groups, possibly because of discrimination they may have faced, tend to mistrust the government which may be exacerbated because of peer effects. This may be an explanatory factor as to why the low uptake we find is highly associated with lower trust. Indeed, among people where trust is not an issue, we find that many ethnic minority populations have a higher willingness towards vaccine uptake, as shown by the results with the interaction terms. An important point to note is this (lack of) trust is not driven by experiences or perceptions of the government’s Covid management but represents their beliefs on institutional trust as seen by their answers to these questions are collated pre COVID. Additionally, the relative unwillingness of those who perceive their financial situation to be poor may reflect a higher opportunity cost of time needed to be vaccinated.

As the UK tries to vaccinate its way out of the pandemic, vaccine hesitancy may prove to be a limiting factor that may prevent the full easing of restrictions. Indeed, trust in government has been an important factor that has affected several decisions made by the government on lockdowns and may have potentially affected its timing and intensity [28]. It has been suggested that lack of trust may well be rooted in historical practices in which minority groups were unethically exploited in medical experiments [19]. This may also explain why younger sections of the BAME population are less hesitant even though their objective risk of facing death or serious illness from the disease is lower. A public health approach that focuses on increasing uptake of vaccination in these at-risk populations must consider both trust that affects willingness to be vaccinated as well as the differential opportunity cost in terms of the expected time away from work for vaccination (including anticipated time off because of side effects) which makes it costly for certain population segments. Improving institutional trust must be combined with making access easier to improve the coverage of all sections of the population. While our analysis is using data from UK, there is suggestive evidence that some of these factors may be global. For example, recent work assessing vaccine willingness in Saudi Arabia shows a far higher rate of hesitancy but suggests people more at risk (older, healthcare workers) or professionals are less likely to express hesitancy and beliefs about health, including perceived side effects from the vaccine are a big driver of hesitancy [29].^.^ A similar study in Bangladesh also found health beliefs rather than socio-economic factors to be the main driver of hesitancy [30]. However, while some of the correlates are similar, these studies cannot be directly compared as they do not include questions on institutional trust.

One of the limitations of our study is the use of survey data conducted until January 2021 as people’s vaccination willingness might have changed with the arrival of information regarding side-effects of the vaccine especially in the case of the AstraZeneca vaccine. Our data reveals that three responses: “I am worried about side effects; I am worried about unknown future effects and I don't trust vaccines” were the main reasons behind vaccine unwillingness (around 60%). When individuals were asked about vaccination willingness, the survey did not ask about the country of the vaccine manufacturer, type of vaccine individuals are likely to be administered, duration of vaccine immunity and place of vaccine administration. During the two rounds of the survey, the UK was going through the second wave of Covid-19 and willingness to take the vaccine might have changed in response to increasing numbers of infections/deaths. Also, the respondents may not have been aware of vaccine efficacy outside clinical trials especially in the context of hospital admissions/severe illness and this could impact COVID-19 vaccine willingness.

## Conclusions

In order to begin the recovery phase of the COVID-19 pandemic, there is an urgency to implement strong and successful global vaccine programmes. However, vaccine hesitancy may derail any intention to do so. Our findings have confirmed previous findings suggesting those from lower socio-economic and minority ethnic communities have the highest rates of vaccine hesitancy. Upon further examination it is clear that this relationship may be partly driven by the lower trust in public sector officials or the government among these communities and socio-economic groups. Therefore, urgent action is needed to promote public health messaging to build trust to encourage improved uptake particularly in groups who are most at risk of negative clinical consequences of COVID-19.

## Data Availability

The data is openly available from https://www.understandingsociety.ac.uk/topic/covid-19
